# Assessment of Non-Response Bias in Estimates of Alcohol Consumption: Applying the Continuum of Resistance Model in a General Population Survey in England

**DOI:** 10.1371/journal.pone.0170892

**Published:** 2017-01-31

**Authors:** Sadie Boniface, Shaun Scholes, Nicola Shelton, Jennie Connor

**Affiliations:** 1 National Addiction Centre, Institute of Psychiatry, Psychology & Neuroscience, King’s College London, London, United Kingdom; 2 Research Department of Epidemiology & Public Health, University College London, London, United Kingdom; 3 Department of Preventive and Social Medicine, University of Otago, Dunedin, New Zealand; Hunter College, UNITED STATES

## Abstract

**Background:**

Previous studies have shown heavier drinkers are less likely to respond to surveys and require extended efforts to recruit. This study applies the continuum of resistance model to explore how survey estimates of alcohol consumption may be affected by non-response bias in three consecutive years of a general population survey in England.

**Methods:**

Using the Health Survey for England (HSE) survey years 2011–13, number of contact attempts (1–6 and 7+) were explored by socio-demographic and drinking characteristics. The odds of drinking more than various thresholds were modelled using logistic regression. Assuming that non-participants were similar to those who were difficult to contact (the continuum of resistance model), the effect of non-response on measures of drinking was investigated.

**Results:**

In the fully-adjusted regression model, women who required 7+ calls were significantly more likely to drink more than the UK Government’s recommended daily limit (OR 1.19, 95% CI 1.06–1.33, P = 0.003) and to engage in heavy episodic drinking (OR 1.23, 95% CI 1.07–1.42, P = 0.004), however this was not significant in men in the fully-adjusted model. When the continuum of resistance model was applied, there was an increase in average weekly alcohol consumption of 1.8 units among men (a 12.6% relative increase), and an increase of 1.5 units among women (a 20.5% relative increase). There was also an increase in the prevalence of heavy episodic drinking of 2.5% among men (an 12.0% relative increase) and of 2.0% among women (a 15.8% relative increase), although other measures of drinking were less affected.

**Conclusion:**

Overall alcohol consumption and the prevalence of heavy episodic drinking were higher among HSE participants who required more extended efforts to contact. The continuum of resistance model suggests non-response bias does affect survey estimates of alcohol consumption.

## Introduction

Non-response bias—where study participants’ answers to questions differ systematically from the potential answers of people who did not agree to take part in a study—has long been recognised as a limitation of surveys that measure alcohol consumption [1]. Heavy drinkers are thought to be more difficult to contact and less likely to respond to surveys [2]. One model that explores how non-response bias may influence survey estimates of alcohol consumption is the ‘continuum of resistance’ model. This assumes that non-participants (non-responders) are similar to each other, and that if they took part in the survey, they would be most similar to the participants who were the most difficult to recruit [3]. The justification is that participants who required many attempts to contact would have been non-participants if the data collection had been stopped earlier, therefore non-participants are more similar to these participants than those who are interviewed after fewer attempts [3]. Better understanding of non-response bias may allow for non-response weights in surveys to be improved. It has also been suggested that it may be preferential to use the continuum of resistance model which takes into account the amount of time and effort required to elicit a response (after Lin and Schaeffer [3]) to adjust for non-response than population weights based on demographics and geographical data [4], which do not necessarily eliminate the bias associated with non-response [5], and may even compound it.

### UK evidence

A recent UK study of risk factors for under-reporting of alcohol consumption was not able to explore non-response bias in detail but recognised there could be value in using the continuum of resistance model in a UK sample in future research [6]. There is limited evidence from the UK but that which exists suggests that alcohol consumption estimates may be biased due to non-response. A 1987 study by Crawford interviewed adults (18+) from three areas in Great Britain about alcohol consumption in their own homes (n = 2,349) [7]. Among men, those who were difficult to contact (requiring 4+ calls) consumed 20.8 drinks a week compared with 16.8 drinks among those who required 1–3 calls (P = 0.02) [7]. In 2002, Lynn and Clarke found that the prevalence of heavy drinking was higher among participants in households that required extended efforts to recruit them (either difficult to contact or who temporarily refused) compared with households that were interviewed after standard efforts [8].

A 2013 study protocol by Gray and colleagues describes how non-response bias can be explored using record linkage [9]. This suggested the downward trend in alcohol consumption in recent years is partially attributable to falling response rates (a trend seen in many health surveys), with fewer heavy drinkers responding over time [9]. Surveys like the Scottish Health Survey (SHeS) use response probability weighting to make them nationally representative, but these weights are based on limited socio-demographic information. The full study published in 2014 found participants in the SHeS experienced lower rates of alcohol-related harm than the general population in Scotland [10]. SHeS participants experienced about two-thirds of the rate of alcohol-related harm compared with the general population (RR 0.69, 95% CI: 0.61–0.76), as well as a lower risk of all-cause mortality (RR 0.89, 95% CI: 0.83–0.96), which was more pronounced in more deprived areas [10]. If the occurrence of alcohol-related harm is higher in the general population than among survey participants, this suggests non-participants may drink more alcohol than survey participants.

### International evidence

Dawson and colleagues [11] recently noted that a number of longitudinal studies have found no difference between those who continue to participate and those who are non-participants at later stages or waves [12, 13], or between early-responding participants and late-responding participants [14, 15]. However numerous other studies have identified important differences between participants and non-participants at later stages [11, 16, 17], or between early responders and late responders or those who required more extensive efforts to recruit [4, 18–21]. There is also evidence from Finland that non-participants are more likely to die from alcohol-related diseases, injuries and poisonings than survey participants [22]. Previous studies using the continuum of resistance model in New Zealand [4] and Canada [18] found higher rates of heavy drinking among late responders, however no studies have used this model in the UK.

In this study we examine how survey estimates of alcohol consumption differ by the number of contact attempts, using the continuum of resistance model. We hypothesise that alcohol consumption will be higher among participants who required more contact attempts. Using the number of contact attempts to distinguish between early- and late-responding participants, we are then able to adjust survey estimates of alcohol consumption for non-response bias using the assumptions of the continuum of resistance model.

## Methods

### Data

The Health Survey for England (HSE) is an annual cross-sectional survey, representative of the adult population (aged 16+) living in private households in England [23]. Full details of the HSE methodology are published annually as reports [24–26]. This study uses three consecutive years of survey data: 2011, 2012 and 2013. Response rates and sample sizes are shown in Table 1, with additional detail available in annual published reports [24–26].

**Table 1:** Response rate by survey year for HSE 2011–13.

Survey year	Individual response rate*	Number of individual adult interviews	Mean (SD) number of contact attempts for participants
2011	59%	8,610	4.27 (2.631)
2012	56%	8,291	4.47 (2.971)
2013	58%	8,795	4.57 (2.899)
Overall	58%	25,696	4.44 (2.840)

*The individual response rate uses the HSE set sample as the denominator. This is the total number of adults at the sampled addresses. Households where at least one adult participated have the number of adults residing in the household recorded as part of the household interview. Interviewers collected information on the number of adults living in households who do not agree to take part in the survey where it was possible to do so. Where households did not provide the number of adults living at the address, the number was estimated based on the mean number of adults in the sampled households where the HSE interviewer was able to establish the number of adult residents.

As in previous years, a computer-assisted personal interview (CAPI) collected information on number of drinking days in the last week and alcohol consumption on the heaviest drinking day in the last week (using a beverage-specific recent recall method), and also on average weekly drinking over the past 12 months (using a beverage-specific quantity frequency method) [27].

In addition to the Health Survey for England dataset that is publicly-available through the UK Data Archive, an additional variable on the number of contact attempts was obtained from the National Centre for Social Research (NatCen) for the purposes of this study. This variable forms part of the HSE paradata set which is held by NatCen.

### Ethics statement

Addresses sampled for the HSE are sent an advance letter and leaflet introducing the survey and stating that an interviewer will be calling to seek permission to interview. It is clear in the advance letter and information leaflet that participation in the survey is entirely voluntary, and that participants may decline to answer individual questions, withdraw or stop at any time, or refuse any particular measurement if they wish to do so. Interviewers will often repeat this information in their introductions and when they are setting up appointments, and throughout the interview as necessary. Individual interviews are conducted with adults who give verbal informed consent. The HSE informed consent procedures, information leaflets and questionnaires are scrutinised by a National Health Service (NHS) ethics committee each year. Ethical approval for the HSE survey years 2011–13 was obtained from the Oxfordshire A Research Ethics Committee: 2011 reference 10/H0604/56, 2012 reference 10/H0604/56, 2013 reference 12/SC/0317. This study is a secondary analysis of previously collected data and so additional ethical approval was not required.

### Analysis

The number of contact attempts for a productive interview ranged from 1–18; however 90% of interviews were completed within 8 contact attempts. The mean number of calls was 4.4 (SD 2.8). We split the number of contact attempts into 2 categories: 1–6 calls (approximately 80% of the sample) and 7+ (slightly under 20% of the sample). Other previous studies have used two groups of respondents in the same way (e.g. [18]). Descriptive statistics assessed demographic and drinking characteristics by the number of attempts to contact (1–6; 7+), with Chi-squared and t-tests to identify differences that were statistically significant. Associations between number of contact attempts and the odds of drinking more than daily and weekly guidelines (as used by the UK Government until January 2016) were modelled using logistic regression, stratified by sex due to the different drinking patterns between men and women. Estimates of alcohol consumption were then adjusted for non-response bias using the probability approach also used by Zhao and colleagues in a 2009 paper [18] and by Rowland and Forthofer in the 1990s [28]. This follows the continuum of resistance model by assuming that non-participants are similar to the survey participants who were most difficult to contact (i.e. 7+ calls). The equation used states that the true prevalence in the population is the sum of the prevalence among participants and non-participants, weighted by the proportions of participants and non-participants:

((prevalenceamongparticipants)*(proportionwhoresponded))+((estimatedprevalenceamongnon-participants)*(proportionwhodidnotrespond))

where the estimated prevalence among non-participants is assumed to be the same as the prevalence among the survey participants who required 7+ calls to elicit a response

All analyses (differences between 1–6 and 7+ contact attempts; and the odds of drinking more than the daily- and weekly-guidelines) were conducted using the HSE interview weight. This weight is computed by NatCen and intends to make the sample representative of the population living in private households in England by adjusting for survey design and demographic predictors of non-response (including sex, household type, geographical area and household social class) [26].

There are two different sources of missing data in cross-sectional surveys such as the HSE. Firstly, individuals living at the sampled addresses who were not surveyed because they did not participate at any stage (unit non-response). We dealt with unit non-response in the present study by using the HSE interview weight described above. The second kind of missing data arises among survey participants, and is due to non-response to certain questions (item non-response). All analyses in the present study used complete-case analysis. That is, in the adjusted regression models, cases were included only if they had non-missing or complete values for all covariates. Missing data in the items of alcohol consumption may have affected the magnitude of the estimates of alcohol consumption shown in the present study–if the item non-response was associated with drinking behaviour. However, item non-response to the items on alcohol consumption was low, and the main focus of the present study was on comparing the estimates of alcohol consumption before and after using the assumptions of the continuum of resistance model. The analyses were conducted in SPSS version 22 (SPSS Inc., Chicago, Illinois, US). Syntax is available on request from the corresponding author.

## Results

Key demographic characteristics are shown by number of contact attempts in Table 2. There was significant variation in the proportion of participants who required 7+ contact attempts to elicit a response by age, economic activity, income, deprivation and region.

**Table 2:** Summary of demographic characteristics of HSE 2011–2013 participants aged 18+ by number of contact attempts.

		Number of contact attempts				
		1–6 calls		7+ calls		*P*-value*
		N	%	N	%	
	Total sample (N = 24,939)	20,240	81.2%	4,699	18.8%	
Sex	Men	9,831	81.0%	2,308	19.0%	
	Women	10,409	81.3%	2,391	18.7%	0.501
Age group	18–34	5,374	75.3%	1,760	24.7%	
	35–54	7,000	78.2%	1,956	21.8%	
	55+	7,867	88.9%	983	11.1%	<0.001
Economic activity	In work	11,199	77.5%	3,254	22.5%	
	Not in work	8,966	86.3%	1,419	13.7%	<0.001
Equivalised income quintile	Lowest	2,939	81.0%	688	19.0%	
	Second	3,115	85.2%	540	14.8%	
	Third	3,139	83.1%	637	16.9%	
	Fourth	3,521	81.5%	797	18.5%	
	Highest	3,300	79.1%	871	20.9%	<0.001
Deprivation quintile	Least deprived	4,171	83.1%	847	16.9%	
	Second	4,436	84.1%	838	15.9%	
	Third	4,221	80.3%	1,037	19.7%	
	Fourth	3,790	78.7%	1,027	21.3%	
	Most deprived	3,623	79.2%	950	20.8%	<0.001
Region	North East	975	77.1%	289	22.9%	
	North West	2,658	80.1%	662	19.9%	
	Yorkshire & The Humber	2,204	87.3%	320	12.7%	
	East Midlands	1,774	82.0%	389	18.0%	
	West Midlands	2,140	81.2%	495	18.8%	
	East of England	2,248	82.3%	485	17.7%	
	London	2,693	73.2%	986	26.8%	
	South East	3,397	83.5%	672	16.5%	
	South West	2,152	84.3%	402	15.7%	<0.001

Weighted using the HSE interview weight.

*P-value comparing two contact attempt groups from Pearson’s Chi-squared analysis

Drinking characteristics by number of contact attempts are shown in Table 3, stratified by sex. There was a greater proportion of men who reported drinking nowadays in the 7+ contact attempts group but the same was not true for women. Drinking more than daily and weekly guidelines and heavy episodic drinking were all more common among men and women in the 7+ calls group than their counterparts in the 1–6 contact attempts group. Compared with those who required 1–6 contact attempts, both men and women in the 7+ contact attempts group had a lower mean number of drinking days but higher average units of alcohol consumed on the heaviest drinking day in the last week as well as higher average weekly alcohol consumption.

**Table 3:** Summary of drinking characteristics of HSE 2011–2013 participants by number of contact attempts.

	Men					Women				
	Number of contact attempts					Number of contact attempts				
	1–6 calls		7+ calls		*P*-value*	1–6 calls		7+ calls		*P*-value*
	N	%	N	%		N	%	N	%	
Whether drinks nowadays—Yes	8,224	81.4%	1,970	83.8%		7,775	73.1%	1,794	73.6%	
No	1,877	18.6%	381	16.2%	0.007	2,865	26.9%	643	26.4%	0.586
Drank more than daily guideline (>4/3 units)–Yes	3,672	36.4%	1,011	56.9%		2,810	26.4%	808	33.2%	
No	6,416	63.6%	1,334	43.1%	<0.001	7,817	73.6%	1,626	66.8%	<0.001
Heavy episodic drinking (>8/6 units)–Yes	2,032	20.1%	571	24.3%		1,253	11.8%	420	17.2%	
No	8,056	79.9%	1,774	75.7%	<0.001	9,373	88.2%	2,015	82.8%	<0.001
Drank more than weekly guideline (21/14 units)–Yes	2,250	22.9%	564	24.9%		1,738	16.8%	450	19.0%	
No	7,587	77.1%	1,700	75.1%	0.038	8,629	83.2%	1,913	81.0%	0.008
	**Mean**	**SD**	**Mean**	**SD**		**Mean**	**SD**	**Mean**	**SD**	
Number of drinking days in the past week†	3.3	2.1	3.0	2.0	<0.001	2.8	2.0	2.6	1.8	<0.001
Units consumed on heaviest drinking day in the last week†	7.4	7.1	8.3	7.7	<0.001	4.8	4.5	5.9	5.2	<0.001
Weekly alcohol consumption (UK units)	14.2	24.1	15.4	23.4	0.043	7.3	15.6	8.3	16.2	0.005

Weighted using the HSE interview weight.

*P-values comparing two contact attempt groups from Pearson’s Chi-squared analysis for categorical variables and from t-tests for continuous variables.

Sample is all adults 18+ who answered the questions about alcohol (24,939 people, with slight variation due to item non response) except for variables marked ‘†’ which are presented only among the adults who reported drinking alcohol in the week prior to interview.

The results of the logistic regression models for drinking more than the weekly and daily guidelines, and heavy episodic drinking, by the number of contact attempts are shown in Table 4. In the unadjusted model for men, there was a borderline increased odds of drinking ‘nowadays’ in the 7+ calls group, however this was not significant in the adjusted models, nor was it significant for women. In all three models, and in both men and women, there were significantly increased odds of drinking more than the daily recommended limit (>4/3 units) in the 7+ calls group, although this was only of borderline significance in the fully-adjusted model for men. For both men and women, there were increased odds of heavy episodic drinking (>8/6 units) in the 7+ calls group in the unadjusted and partially-adjusted models, however in the fully adjusted model this was only significant for women. There were no significant associations between the number of contact attempts and drinking more than the weekly guidelines (>21/14 units).

**Table 4:** Odds ratios (OR) and 95% confidence intervals (CI) for drinking more than weekly and daily guidelines and heavy episodic drinking by number of contact attempts (weighted sample).

	Number of contact attempts	Unadjusted			Partly adjusted^a^			Fully adjusted^b^		
**Men**		**OR**	**95% CI**	***P*-value**	**OR**	**95% CI**	***P*-value**	**OR**	**95% CI**	***P*-value**
Whether drinks nowadays	1–6 calls	1.00			1.00			1.00		
	7+ calls	1.16	1.00–1.34	0.048	1.16	1.00–1.34	0.051	1.14	0.98–1.34	0.089
Drinking more than daily guidelines^1^	1–6 calls	1.00			1.00			1.00		
	7+ calls	1.24	1.12–1.38	<0.001	1.14	1.03–1.27	0.012	1.12	1.00–1.24	0.050
Heavy episodic drinking^2^	1–6 calls	1.00			1.00			1.00		
	7+ calls	1.28	1.13–1.44	<0.001	1.14	1.01–1.29	0.036	1.10	0.97–1.24	0.141
Drinking more than weekly guidelines^3^	1–6 calls	1.00			1.00			1.00		
	7+ calls	1.08	0.96–1.22	0.202	1.12	0.99–1.26	0.073	1.09	0.97–1.24	0.156
**Women**		**OR**	**95% CI**	***P*-value**	**OR**	**95% CI**	***P*-value**	**OR**	**95% CI**	***P*-value**
Whether drinks nowadays	1–6 calls	1.00			1.00			1.00		
	7+ calls	1.03	0.91–1.16	0.632	0.97	0.86–1.10	0.672	0.96	0.85–1.10	0.575
Drinking more than daily guidelines^2^	1–6 calls	1.00			1.00			1.00		
	7+ calls	1.36	1.22–1.51	<0.001	1.22	1.10–1.36	<0.001	1.19	1.06–1.33	0.003
Heavy episodic drinking^3^	1–6 calls	1.00			1.00			1.00		
	7+ calls	1.47	1.28–1.68	<0.001	1.25	1.09–1.44	0.002	1.23	1.07–1.42	0.004
Drinking more than weekly guidelines^1^	1–6 calls	1.00			1.00			1.00		
	7+ calls	1.11	0.97–1.26	0.126	1.07	0.94–1.22	0.317	1.05	0.92–1.21	0.452

Abbreviations: OR: odds ratio; CI: confidence interval

^1^Drinking more than 4/3 units on heaviest drinking day in the last week.

^2^Drinking more than 8/6 UK units on heaviest drinking day in last week.

^3^Drinking more than 21 (men) / 14 (women) UK units a week.

^a^Adjusted for age.

^b^Adjusted for age, economic activity, income, deprivation and region.

Under the continuum of resistance model to include non-participants in survey estimates (by assuming that non-participants are similar to the survey participants who were the most difficult to contact), the estimated quantity and frequency of alcohol consumption changed by varied amounts in relation to the estimates of alcohol consumption produced using the survey weights alone (Table 5). The majority of estimates increased, with the largest percentage increases in the prevalence of heavy episodic alcohol consumption (with relative increases of 12.0% for men and 15.8% for women, and absolute increases of 2.5% for men and 2.0% for women); and increases in average levels of weekly alcohol consumption (with relative increases of 12.6% for men and 20.5% for women, and absolute increases of 1.8 units a week for men and 1.5 units for women). The number of drinking days in the last week decreased by a small amount, and this is consistent with a more hazardous drinking pattern.

Finally, if self-reported consumption was compared with alcohol sales for the same time period (which is equivalent to 19.1 units per week per adult aged 16+ when averaged across financial years 2011–12 and 2012–13) [29], the estimates adjusted under the assumption of the continuum of resistance model represent a substantial increase in alcohol sales coverage. Coverage increases from 57.0% as captured by self-reported consumption using the HSE weighted estimate (where mean weekly alcohol consumption among all adults was 10.9 units a week), to 66.4% using the assumptions under the continuum of resistance model (where mean weekly alcohol consumption among all adults was 12.7 units a week).

**Table 5:** Population alcohol consumption estimates assuming non-participants are similar to participants who were difficult to contact.

	Unadjusted estimates	Adjusted using survey non-response weights*	Further adjusted under the assumption of the continuum of resistance**	Difference	Percentage change
**Men**					
Prevalence of drinking nowadays	82.4%	81.9%	82.7%	0.8%	1.0
Drinking days in last week, among drinkers (mean number)	3.3	3.2	3.1	-0.1	-3.3
Alcohol consumed on heaviest drinking day in the last week (mean units)	7.2	7.6	7.9	0.4	5.0
Prevalence of drinking above daily guidelines (>4/3 units)	37.0%	37.7%	37.7%	0.0%	-0.1
Prevalence of heavy episodic drinking (>8/6 units)	19.8%	20.9%	23.4%	2.5%	12.0
Average weekly alcohol consumption (mean units)	14.7	14.4	16.3	1.8	12.6
Prevalence of drinking above weekly guidelines (>21/14 units)	23.9%	23.3%	25.3%	2.0%	8.7
**Women**					
Prevalence of drinking nowadays	73.5%	73.2%	73.4%	0.2%	0.2
Drinking days in last week, among drinkers (mean number)	2.8	2.8	2.7	-0.1	-3.0
Alcohol consumed on heaviest drinking day in the last week (mean units)	4.9	5.0	5.4	0.4	7.5
Prevalence of drinking above daily guidelines (>4/3 units)	27.7%	27.7%	27.7%	0.0%	-0.1
Prevalence of heavy episodic drinking (>8/6 units)	12.4%	12.8%	14.8%	2.0%	15.8
Average weekly alcohol consumption (mean units)	7.6	7.5	9.0	1.5	20.5
Prevalence of drinking above weekly guidelines (>21/14 units)	17.3%	17.2%	17.9%	0.7%	4.1

‘Difference’ column relates to the difference between the weighted survey estimates and continuum of resistance estimates.

*Population weights are computed by NatCen and they make the sample representative of the population living in private households in England by adjusting for survey design and standard demographic and regional predictors of non-response.

**The continuum of resistance assumes participants who took 7+ calls to complete an interview have similar alcohol consumption to non-participants.

## Discussion

### Key findings

This study found participants in the Health Survey for England who took more attempts to contact have different socio-demographic characteristics and different drinking patterns than participants who took fewer attempts to contact. Most notably, the prevalence of heavy episodic alcohol consumption, and levels of average weekly alcohol consumption were higher for those who took more attempts to contact, although other measures of alcohol consumption showed less variation by the number of contact attempts. The measures of alcohol consumption which increased the most when the estimates were further adjusted according to the assumptions of the continuum of resistance model were measures most associated with acute harms (i.e. heavy episodic drinking) and chronic harms (i.e. total weekly consumption). The decrease in the average number of drinking days per week is consistent with a more hazardous pattern, since it suggests higher volumes are being consumed on each occasion. Our findings lend support to extended efforts to recruit and follow up participants in research studies in order to reduce bias in estimates of drinking.

### Strengths

To our knowledge, this is the first study using the assumptions of the continuum of resistance model to assess non-response bias in survey estimates of alcohol consumption in the UK. This also represents a unique use of existing data: the HSE is publicly available through the UK Data Archive, and the paradata (including data on the number of contact attempts) is routinely collected by NatCen, and is available from NatCen on application to their Data Release Panel.

We used three consecutive years of a large, nationally representative survey (the HSE) which is the source of some of England’s national statistics on alcohol drinking. The response rate for the HSE is in line with other similar surveys (for example the SHeS), therefore it may be that similar findings apply in other populations where drinking patterns are similar.

### Limitations

It is difficult to compare the findings of this study with other studies. This is because the definition of the number of contact attempts or the amount of time taken to respond to an invitation varies from study to study due to sampling and recruitment methods employed and different measures used. However we would say that our findings are broadly similar to those of Meiklejohn 2012 [4] and Zhao 2009 [18]. Our findings also corroborate those of work conducted in Scotland, which used record linkage to examine rates of alcohol-related harm in participants in the SHeS compared with the general population [9, 10]. In this record linkage study, the difference in mortality rates between survey participants and the general population was used to investigate the validity of survey estimates, whereas in the present study we have estimated the potential impact of non-response on the survey estimates of consumption.

Total number of contact attempts is not an ideal measure of willingness to take part in a survey. In some instances it can be beneficial if the interviewer leaves and returns on another occasion so as to avoid a refusal [8]. Often, a substantial proportion of the total number of calls may take place after initial contact has been established, where the interviewer is trying to find a suitable time to complete the survey. For example in Lynn and Clarke’s 2002 study, fewer than half of households that received 10 or more calls required 10 calls to make an initial contact, the remainder required the extra calls subsequent to the first contact being established [8].

Missing data is a perennial problem for the analysis of survey data. In the present study we used the HSE interview weight, and the assumptions under the continuum of resistance model, to correct for unit non-response: thereby assigning larger weight to the subgroups of the population with lower propensities to respond to social surveys. However, our use of complete-case analysis may mean that there may still be residual bias in our estimates of alcohol consumption due to item non-response; for example, it is possible that the subset of survey participants with missing values for the alcohol questions may have been heavier drinkers than the participants with non-missing alcohol data.

Finally, the use of 7+ contact attempts as a measure of ‘difficult’ to contact is an arbitrary cut point (although this was also used by Zhao and colleagues [18]). Almost 20% of our sample required 7+ calls to elicit a response. We believe this was an appropriate proportion for reasons of obtaining adequate statistical power, and we did identify important differences between the 1–6 and 7+ groups. In a 2014 US study by Messiah and colleagues it was suggested that five attempts to contact was sufficient to reduce selection bias. After five attempts, the return per visit was low, and the prevalence of seven health conditions studied changed only by a very small amount, suggesting additional attempts were of decreasing value [30]. Further research could identify the optimum number of contact attempts, however this was beyond the scope of the present study.

### Future research

There are other variables held by NatCen in the HSE paradata which could also be of interest in relation to how drinking is measured in surveys. For example, there is a variable describing the interviewers’ assessments of overall data quality, and other measures of ‘cooperativeness’, such as consent to record linkage and consent to a subsequent nurse visit.

Future research could address the limitation of the complete-case analysis used in our study by examining the impact on the estimates of alcohol consumption by using the technique of multiple imputation to substitute values for a missing items on alcohol consumption, alongside the adjustment made in this study under the assumptions of the continuum of resistance model (utilising the HSE paradata to distinguish between early- and late-responding participants). The continuum of resistance model could also be used to explore other health behaviours such as physical activity, and there is potential for the number of contact attempts to be incorporated as a covariate in the models used to estimate the survey non-response weights (such as the HSE interview weight). It would also be valuable to explore the continuum of resistance model in the context of a different study design, for example in follow ups to a randomised controlled trial in order to see any similarities in the patterns observed with the HSE.

## Conclusion

Using the assumptions of the continuum of resistance model to further adjust for potential non-response bias in survey estimates of alcohol consumption, there was a 12.6% relative increase in average weekly alcohol consumption (absolute increase of 1.8 units a week) in men and a 20.5% relative increase in women (absolute increase of 1.5 units a week). There was also a 12.0% relative increase in the prevalence of heavy episodic alcohol consumption (absolute increase of 2.5%) in men and a 15.8% relative increase among women (absolute increase of 2.0%). Other measures of drinking were less affected. This study provides evidence that non-response bias differentially affects survey estimates of alcohol consumption in a nationally-representative sample in England.
